# Multilevel survival analysis of health inequalities in life expectancy

**DOI:** 10.1186/1475-9276-8-31

**Published:** 2009-08-23

**Authors:** Min Yang, Sandra Eldridge, Juan Merlo

**Affiliations:** 1 Centre for Psychiatry, Barts and The London, Queen Mary's School of Medicine and Dentistry, William Harvey House, 61 Bartholomew Close, London EC1A 7BE, UK; 2 Centre for Health Sciences, Barts and The London, Queen Mary's School of Medicine and Dentistry, Abernethy Building, 2 Newark Street, London E1 2AT, UK; 3 Social Medicine, Lund University MAS, CRC, Ing 72, Hus 28, Plan 12, 205 02 MALMÖ, Sweden

## Abstract

**Background:**

The health status of individuals is determined by multiple factors operating at both micro and macro levels and the interactive effects of them. Measures of health inequalities should reflect such determinants explicitly through sources of levels and combining mean differences at group levels and the variation of individuals, for the benefits of decision making and intervention planning. Measures derived recently from marginal models such as beta-binomial and frailty survival, address this issue to some extent, but are limited in handling data with complex structures. Beta-binomial models were also limited in relation to measuring inequalities of life expectancy (LE) directly.

**Methods:**

We propose a multilevel survival model analysis that estimates life expectancy based on survival time with censored data. The model explicitly disentangles total health inequalities in terms of variance components of life expectancy compared to the source of variation at the level of individuals in households and parishes and so on, and estimates group differences of inequalities at the same time. Adjusted distributions of life expectancy by gender and by household socioeconomic level are calculated. Relative and absolute health inequality indices are derived based on model estimates. The model based analysis is illustrated on a large Swedish cohort of 22,680 men and 26,474 women aged 65-69 in 1970 and followed up for 30 years. Model based inequality measures are compared to the conventional calculations.

**Results:**

Much variation of life expectancy is observed at individual and household levels. Contextual effects at Parish and Municipality level are negligible. Women have longer life expectancy than men and lower inequality. There is marked inequality by the level of household socioeconomic status measured by the median life expectancy in each socio-economic group and the variation in life expectancy within each group.

**Conclusion:**

Multilevel survival models are flexible and efficient tools in studying health inequalities of life expectancy or survival time data with a geographic structure of more than 2 levels. They are complementary to conventional methods and override some limitations of marginal models. Future research on determinants of health inequalities in the LE of the specific cohort on the household and individual factors could reveal some important causes over the marked household level inequalities.

## Background

The health status of individuals is determined by the interactive effects of multiple genetic, environmental, and socio-demographic factors operating at different levels, such as individuals' micro level socio-economic status and lifestyles, together with the macro level characteristics of the public health system and their residing geographical areas. As a result, health outcomes such as mortality rates and life expectancy are different in different population subgroups. Measuring health inequalities plays a fundamental role in the assessment of the performance or achievement of a health system, as part of the decision-making process that leads to amenable intervention. Although health inequalities have often been interpreted as a variation in health status measured by a specific health outcome across subgroups within a population [1], to reflect the sources of health determinants, a good health system should be measured against two objectives: the best attainable average level of health in the population it serves and the smallest feasible differences among individuals or groups within that population [2]. Most health inequality studies have focused on average differences across groups of people; addressing health inequalities caused by economic [3], social class [4,5], demographic [6] and educational [7] diversity, but additional understanding about how variation in health is distributed at different levels may provide relevant information for public health intervention [8].

Given the multi-level influences on health, analyses at only the individual-level may ignore the potentially important effects of higher level factors. However, analyses of data aggregated to geographic levels may suffer from ecological bias [9] and has been criticised for not adequately addressing health inequality [1]. Analyses that can account for effects at different levels are key.

The World Health Organisation (WHO) proposed assessing the performance of national health systems using individual level health inequalities alongside the average level of health in a population [3,10,11]. In an analysis of individuals' health inequality based on mortality, Gakidou [12] proposed a measure of total health inequality derived from the beta-binomial regression model, which unified treatment of various measures including the Gini coefficient [13] and other estimates of inequalities. Their method has been used to estimate the risk of death in children under two and the between-individuals variance in the risk, based on a two-level data structure of children nested within families. The model provided absolute and relative inequality measures compared across 50 countries. Their model was adequate for mortality outcome based on either cross-sectional survey or cohort data with death event observed for all individuals in the cohort. For time to death or survival time as an outcome with censored time for some individuals based on cohort design, the beta-binomial model will not work. These researchers instead applied the frailty survival model to estimate the risk of death distribution in adults [14] where 'frailty' refers to excess systematic variability or random effects due to unmeasured factors at the community or geographic level which were not explained by individual-level covariates in the model. Having controlled for community-level effects, some inequality indices were also derived and compared between gender and age groups. These marginal models are flexible enough to model distribution of health outcomes with adjustment of individual level confounding, and can provide efficient estimates of health outcomes by accounting for overall variation at higher levels. However, these models do not separate the total variance explicitly by the source of variation, and cannot fit data with more than two levels of hierarchy [15,16].

In practice, a model with a hierarchical structure of two or more levels is often plausible such as having individuals nested within households within residential areas. Inequality estimates which do not take account of this hierarchical structure may not identify variation at relevant levels and thus fail to pinpoint potential targets for interventions [8].

In recent years, multilevel models (MLM) have been available to further improve analysis of health inequalities over the marginal models [15,17]. By explicitly modelling separate random effects at each level of the data, the MLMs can provide estimates of the outcome distribution at each level, quantify and distinguish contextual effects (at higher levels) from individual effects and separate variances of social groups from the total variation within a population [15]. The importance of MLM analysis is well recognized in social sciences, public health, health care [18] and epidemiological research [19]. The use of MLM in health inequalities research is increasing [8,18,20], but most previous reports are on multilevel logistic models for mortality outcome based on cross-sectional data. There is a lack of reports on multilevel models directly for modelling life expectancy (LE) based on cohort data with censored information on survival time, although multilevel survival models [15,21] built in the software MLwiN [17] are already available.

In this study we apply multilevel survival models to investigate distributions in life expectancy (from 1970 onwards) among individuals from different socio-economic sub groups and multiple sources of variation of life expectancy in a cohort of elderly Swedish individuals followed up for 30 years. Like any multilevel models, multilevel survival time models consist of two parts, fixed effects and random effects. The model estimates of the fixed effects measure the mean differences between the socio-economic sub groups in life expectancy (so called socio-economic inequality), and estimates of the random effects quantify the variance or stochastic elements in life expectancy that cannot be accounted for by the mean differences. The total variance in life expectancy is disentangled into components for variation between municipalities, between parishes, between households and between individuals. The unexplained heterogeneity in the term of model residuals then is used to calculate some established and meaningful inequality indices for the health outcome of LE at different levels in the data hierarchy.

Our study is mainly aimed at introducing the new method for health inequality research. We illustrate the methods using a real example analysis that explores socio-economic and geographical inequalities in life expectancy in the elderly Swedish cohort. The analysis has four specific aims: (i) to identify components of the total variation in individual life expectancies by the geographic level of their residence, namely, individual, household, parish, and municipality; (ii) to explore the extent to which socio-economic and demographic factors defined at different levels can account for the variation in life expectancy; (iii) to compare the variation in life expectancy within socio-economic and demographic groups within the population; (iv) to explore absolute and relative indices of health inequalities in life expectancy in different population subgroups.

## Methods

### Study population

This study is based on the LOMAS (Longitudinal Multilevel Analysis in Scania) - a record linkage database that includes all the individuals living in Scania, Sweden, during the period 1968 to 2006. Scania is the most southern part of Sweden and contains approximately 12% of the Swedish population. The project has been approved by the Regional Ethical Committee in South Sweden. LOMAS was assembled with the allowance and assistance of Statistics Sweden, The National Board of Health and Welfare (Centre for Epidemiology), and the Region of Scania (Unit of Social Medicine). A unique ten-digit personal identification number assigned to each person in Sweden was used by the Swedish authorities to link the different registers. However, the research database does not contain the real personal identification number of the individuals but rather an encrypted number that ensures the anonymity of the individuals. Our investigation uses information from the 1970 Swedish Census, the 1970 Population Register, and the Mortality Register for the period 1970-2000.

In the present analysis we defined a baseline cohort composed of all 49,154 individuals aged 64 to 69 and residing in Skåne by 31^st ^December 1970. The dataset consists of 42,838 households in 402 parishes within 69 municipalities of the Scania region; thus providing four levels of hierarchy. We followed individuals from baseline until death or end of follow-up by 31^st ^December 2000. Over 90% of individuals in this cohort had completed the follow-up with outcome events (death) observed, with 7.6% of males and 5.4% of females censored.

Since our study is a pure cohort study, migrants into the study region after 1970 are not part of the study sample. We assumed a very low mobility of the elderly population in general, and thus assumed that the number of such migrants would be very small. Initial cohort members who migrated within the study region, or even within the country, are included because their death should have been registered and kept in the regional or national databases. Those who emmigrated to other countries after 1970 are treated as censored at the time of study. The proportion of censoring data was 1.2% to 4.7% for men and 3.4% to 12.3% for women among five age groups. The proposed survival time models are designed to handle such data.

### Predictors of life expectancy

Age and gender are individual level variables. We categorized age at baseline in 1970 into five categories of one year (i.e. 65, 66, 67, 68 and 69) and used 65 years as reference in the comparisons.

Socio-economic status was defined at the household and municipality level. The categorisation at each level was based on descriptive pattern analysis of the data to find threshold of variables that more or less maximised differences between categories. At the household level, we defined a variable of 4 category levels by combining household monthly disposable income per head of family members and family size to represent household socioeconomic position (Household SEP). The poorest were families that had more than 4 members without any disposable income. The next poorest were families that had 1-4 members without any income. Moving on to the higher level were families with income between 1 - 1000 SEK per family member, and the top group were those with more than 1000 SEK per family member. For the socioeconomic characteristics of the municipalities we used the median individual income of each municipality in 1970 as a municipality level variable.

### Statistical methods

Multilevel accelerated failure time models fit time of death to individuals, taking into account attributes of the time until death at both individual and context levels [15,22]. Let *T *indicate time to death, the simplest form of a 2-level model can be expressed as *y *= log (T) = *β*_0 _+ *v*_2 _+ *v*_1_, with a fixed parameter estimate *β*_0 _= log (*t*_0_) the logarithm of the median life expectancy without adjusting for confounding and two random variables *v*_2_, *v*_1 _to reflect those between and within individual variance around the median life expectancy respectively. In other words, the median life expectancy varies between areas where individuals resided and between individuals within an area. The variance of the two terms is the estimate of between and within individual variance  and  respectively. The model with log link assumes independence of the two random variables in log scale and hence their variances are additive and sum to total variance of log(LE) [15]. For data of more than 2 levels in our case with municipalities at level 4, parishes at level 3, household at level 2 and individuals at level 1, the total variance can be further disentangled explicitly to estimate the variance at each level. The model is extended by adding two more random parameters so that the total variance of individual life expectancy in log scale has four components and sums to . In the situation where a large proportion of households consists of only one individual, variance components can be constructed to reflect the data structure so that the level 2 variance  measures only variability across those households with more than one individual.

To adjust for confounding or estimate average inequality of subgroups such as age, gender or income level; the model can be further extended in any regression model by adding these variables as covariates. Further more, distributions of life expectancy by gender for example, can be specified by the mean and variance together for men and women respectively, using a variance partitioning model [22]. This provides us with a full picture of the health inequality of a subgroup at both the average level and variability at the individual level.

Given the observed lifetime of individuals, the outcome variable, the estimated median life expectancy of the study population would be  and  for the *i*^th ^individual with covariate X. The survival probability of the *i*^th ^individual is the cumulative probability function of the distribution, for example,  for log-Normal distribution.

Indices of so called relative and absolute health inequalities [2,12] for life expectancy can be derived based on model estimates. For Individual-Mean differences, IM [a,b] = , where *a *is the power order of the numerator and *b *that of the denominator, μ the population mean of the inequality variable *y *and *n *the population size. When both *a *and *b *are equal to 2, i.e. the quadratic term for both the numerator and denominator, the classical Coefficient of Variance (CV), an index of relative health inequality, applies, and , where n is the total number of individuals,  the parameter estimate for population mean in association with a subgroup in the model, and  the standard error estimate at the individual level 1. The IM can be calculated for individuals in different groups such as gender or household socioeconomic groups. Replacing  with ,  with , we obtain an estimate of the relative inequalities among parishes.

At the individual level, we can consider Inter-Individual differences, indicated as , and the specific index of so called absolute inequalities but relative to the population mean  is in theory comparable with the traditional Gini coefficient in the single level case. When the numerator takes a cubic term, i.e. *a *= 3 or II[3,1], the indices weigh towards the extreme individuals. Here  is the survival probability estimated from the multilevel model including the level 1 random effects of *v*_1_. For comparison purpose we also calculated the traditional Gini coefficient [13] for inter individual differences in their survival probability and inter area differences in parish LE based on both raw data and model estimates. For the latter, the estimated median life expectancy of parish mean is the y variable (*M*_*v*3_). For example, for the inter-parish inequalities, the median life expectancy is estimated as .

To address the four objectives of the study, three steps of analysis were carried out. At Step 1 for objective (i), we fitted a series of models with random effects included for 2 or 3 or all 4 levels in order to identify the variance components of random effects that best fitted the pattern in data. All models included sex and age of individuals in 1970 as covariates. For each model we tested the significance of the variance components using the Wald statistic [15]. This analysis enabled us to assess how much of the variation in individuals life expectancy was attributable to each of the four levels and to establish a basic variance component model in which the variation of life expectancy can be estimated at each level.

At Step 2 for objective (ii), we examined the extent to which the household socio-economic group affects individuals' health by adding the household socio-economic variable into the basic model established in Step 1. The differences in the median life expectancy between individuals from different family socio-economic groups are captured by the new regression coefficients in the model, controlled for age and sex. The differences in the estimated variances at each level between the new model including the household SES variable and the baseline model without the variable reflected the variability in life expectancy explained by the variable only at either individual level or higher levels. Other risk factors potentially confounding the relationship between household SES and individual mortality could have been included in the model to illuminate relationships further, but in the interests of presenting a simple model for illustrative purposes, we have not pursued such an analysis.

For objective (iii), we then estimated variance in life expectancy for men and women and for each household SES group in order to compare the distribution of LE among the subgroups.

Finally, in Step 3 for objective (iv), we calculated the indices of relative and absolute health inequalities based on our models, and compared them with traditional coefficient of variance (CV) and Gini coefficient.

We also assessed the validity of our models by comparing the age-gender specific life expectancy in remaining years estimated by our models to those by the Kaplan-Meier (KM) estimator based on the raw data.

## Results

Table 1 shows crude mortality rates and the KM estimator of LE for life remaining after 1970 by age in 1970, gender, municipality and household socio-economic status. Men, but not women, tend to live longer in municipalities with lower incomes. There is a strong gradient effect of household socio-economic level with LE for both men and women, with shorter LE for those from large families on low incomes.

**Table 1:** Mortality (%) and median LE of life remaining (KM estimator) by gender, Skåne in Sweden, 1969-2000

**Variables**	**Males**		**Females**	
		
	**N (%)**	**LE (SE), year**	**N (%)**	**LE (SE), year**
Age in 1970				
65	4940 (95.3)	15 (0.17)	5685 (87.7)	19 (0.15)
66	4791 (96.3)	14 (0.16)	5517 (90.9)	19 (0.15)
67	4527 (97.6)	13 (0.16)	5221 (92.9)	18 (0.15)
68	4336 (98.1)	12 (0.16)	5117 (95.0)	17 (0.15)
69	4086 (98.8)	12 (0.16)	4934 (96.6)	16 (0.15)
Log-rank Chi-square (P value)		445.6 (<0.0001)		685.2 (<0.0001)
Municipality Income (SEK)				
1000 - 2000	6737 (96.7)	14 (0.13)	6850 (92.5)	18 (0.13)
2001 - 2150	5234 (97.1)	13 (0.15)	5956 (92.1)	18 (0.14)
2151 - 2300	3587 (97.4)	13 (0.17)	4402 (93.3)	17 (0.15)
> 2300	7122 (97.4)	12 (0.13)	9266 (92.1)	18 (0.12)
Log-rank Chi-square (P value)		71.6 (<0.0001)		9.63 (0.022)
Household Socioeconomic Level				
Poorest (family size > 4 & income = 0)	223 (96.4)	9 (0.66)	214 (98.1)	12 (0.71)
Poor (family size 1 - 4 & income = 0)	851 (98.0)	11 (0.35)	1218 (93.8)	16 (0.39)
Middle (income: 1 - 1000 per head)	15113 (97.2)	13 (0.09)	17914 (92.9)	18 (0.08)
Top (income > 1001 per head)	6493 (96.6)	14 (0.14)	7128 (90.8)	19 (0.13)
Log-rank Chi-square (P value)		103.8 (<0.0001)		211.4 (<0.0001)
Total	22680 (97.1)	13 (0.07)	26474 (92.4)	18 (0.07)

Note: The χ^2^values (df = 1) of linear trend test for Age in 1970, municipality income and household SES group are 1115.0 (p = 0.000), 20.7 (p = 0.000) and 267.5 (p = 0.000) respectively, controlling for gender.

To obtain model based age and gender specific LE for model validation, Model C (Additional file 1, Table S1) which includes age and gender was considered. Adding the three random part estimates to the fixed part estimates in Model C, we obtained the model based median life expectancy of males for the age cohorts 65, 66, 67, 68 in 1970 as 14.4, 13.8, 13.2, 12.2 and 11.8 respectively, and of females as 19.1, 18.2, 17.4, 16.3 and 15.5 accordingly. They were fairly close to the K-M estimators, suggesting acceptable fitting to the data.

### Variation in life expectancy attributable to each of the four levels

Additional file 2, Table S2 shows that ignoring parishes and households, the 2-level model revealed a small proportion (6.4%) of total variance attributable to municipality. However, the 3-level model suggested that over 90% of variation seemingly captured at the municipality level in the 2-level model was mainly due to differences between parishes. Moreover, the parish variance was further absorbed by household differences that explained about 18% of total variation in LE of the cohort. We need to bear in mind that the amount of household variation was only contributed by 15% of families with more than one member. Much greater variation between families could have been observed, had the cohort consisted of more large families. Finally, the 4-level model showed that variation in life LE in the older Swedish cohort was predominantly due to individual differences, followed by household differences. Although remaining significant statistically, the variability among parishes was small. The effects of municipality on the variance of LE in total can be ignored.

Further analysis in this study was based on the 3-level structure assuming no random effects or stochastic components in LE among municipalities.

### Differences in life expectancy by gender and income

In Additional file 1, Table S1, models A and B estimate the mean differences in life remaining beyond 1970 between genders and household socio-economic levels respectively, ignoring differences in age and area income. Models C to E estimate differences in gender, or household socio-economics, or age group or area income, after controlling for each other factor. Based on model E, the LE (after 1970) of men lived in municipalities of the average income in the study cohort is only about 74% of that of women in municipalities of the same income level. The LE in men was reduced further by 0.59 of women's mean LE as the area income increased by one unit. This could be due to lower income inequalities in lower income municipalities, which affected men more than women. In the study cohort 14.4% more single men and 47.9% more single women lived in the top income area (>2300 SEK) than the lowest income area (1000-2000 SEK). It could be hypothesized that single men in higher income areas had fewer social networks to help them cope with lonliness and might have lower life quality than family men in lower income areas. Women are generally better than men at making networks when they live alone. Our study focused on the modelling and did not further test the hypotheses behind this finding.

The mean differences in life expectancy in the four household SES categories suggest significant linear effects in the logarithm scale or exponential effects in the raw scale. Compared to the poorest group, the median LE was about 1.41 times, 1.64 times and 1.78 times longer for individuals in the income groups 'poor', 'middle' and 'top' respectively. Comparing between Models C and D, the additional effects of the household socio-economic level explain only 1.8 percent of the variance among households and a large amount of variation remains among individuals. This suggests that household socio-economic level measured mainly by income per family member could not be an attribute to the large variation in the individuals' LE among families, although the large difference in this health outcome between household income within subgroups was important in health intervention.

Municipality income has no significant effect on women's LE but shows a negative effect on men's (Model E).

### Variances in life expectancy by gender and by household poverty

For variability of life remaining or survival time, the raw coefficient of variance (CV) demonstrates greater variation of survival time in men than in women (Table 2), and a graded pattern of reduced variation of the LE followed by the decreasing of household poverty level of individuals. The adjusted variance based on the model E in Additional file 1, Table S1 has presented comparable estimates of variation for men and women (0.569 and 0.446 respectively), and similarly graded changing pattern for subgroups by the household socio-economic level.

**Table 2:** Health inequality measures by gender and household SES from raw data and from 3-level models, Skåne in Sweden, 1969-2000

	**Measures Based on Raw Data**			**Measures Based on Model E**		
		
	K-M LE (SE)	Gini Coef.	CV^a^	Variance (SE)	II[1,1]	√IM[2,2]
**Household SES**						
Poorest	11.0 (0.50)	0.419	0.655	0.856 (.062)	0.168	0.394
Poor	14.0 (0.27)	0.362	0.562	0.620 (.033)	0.141	0.296
Middle	15.0 (0.07)	0.320	0.511	0.502 (.031)	0.125	0.252
Top	16.0 (0.10)	0.304	0.490	0.479 (.031)	0.117	0.239
**Gender**						
Men	13.0 (0.07)	0.318	0.552	0.569 (.004)	0.237	0.376
Women	18.0 (0.07)	0.317	0.454	0.446 (.004)	0.217	0.289

^a ^

**Figure 1:**
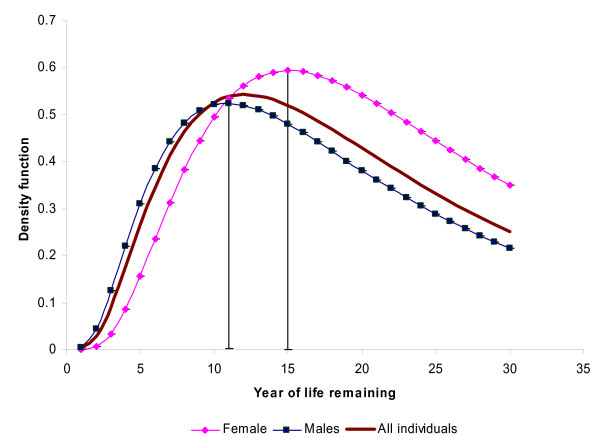
**Density function of survival year in life remaining by gender based on model estimates, Skåne in Sweden, 1969-2000**.

**Figure 2:**
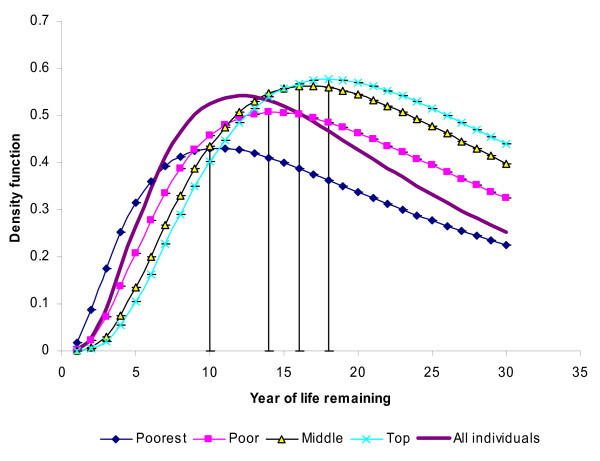
**Density function of survival year in life remaining by household socio-economic category based on model estimates, Skåne in Sweden, 1969-2000**.

Derived from the model estimates, Figure 1 presents density functions or the distribution of life remaining by gender, and Figure 2, by household socio-economic groups. The vertical lines in the figures represent the median years of life remaining for different categories of each variable.

### Relative and absolute indices of inequality

The absolute health inequality index of Inter-Individual difference (II[1,1]) by household socio-economic level, calculated from the model estimates, has shown a similar pattern as the raw Gini's coefficient (Table 2), which suggests that among the elderly population in Sweden health inequalities are larger for those in poorer households. Although women have longer survival time or LE after 1970 on average than men in the cohort, both Gini coefficient from the raw data and the absolute inequality index by II[1,1] from our model demonstrate significant differences between genders.

As shown in Table 2, the model-based relative inequality index of the Individual-Mean difference IM[2,2] presents a similar pattern to the conventional coefficient of variance (CV) based on raw data. Those individuals from the poorest households showed the largest relative inequalities, whilst those from the richest showed the smallest relative inequalities. Males tended to have a larger coefficient of variation or relative inequality compared to females in this age cohort.

### Inequalities at Parish level

Assessing health inequalities of life expectancy at area level would be of much practically interest for policy making and intervention by health authorities. It would be important to show how area health inequalities in life expectancy could be influenced by possible determinants of risk factors at macro or micro level or both, and what the area inequality level is after adjusting for those factors. The model based approach enables us to conduct such an investigation efficiently. In Table 3 we presented three inequality measures for parish life expectancy derived from three models and compared them with the ecological analysis which was simply based on parish median LEs. The inequality measures based on the 3-level random effect survival models were adjusted respectively for gender alone (Model A), for age alone (Model B) and for age, gender, household SES and area income all together (Model E). They were also exclusive of individual and household random effects. In principle, more adjustments are included in the model, less variation is in LE to be captured by the inequality measures derived from the model. However, the expected pattern was not observed in both the Gini coefficient and the CV among the models. This was due to the fact that there was little variation in LE, as seen in the variance estimate in Additional file 1, Table S1, between parishes after separating it from the individual and household heterogeneity by the 3-level model. All measures from the simple ecological analysis are larger than model based estimates, because that the ecological analysis embedded in a single measure variance of all sources and variability that could have been explained by individual age or gender or household SES or area income. Although the ecological analysis could be stratified by gender, age or household SES in examining associates or intervention effects of health inequalities, statistical power will be reduced as the group of stratification increases. In contrast, the model based approach retains all data in the analysis, hence is more efficient, flexible and powerful for complex data in studying health inequalities.

**Table 3:** Parish inequalities in life expectancy (n = 401), Skåne in Sweden, 1969-2000

	**Ecological analysis**	**Model A**	**Model B**	**Model E**
Gini coefficient	0.090	0.014	0.015	0.013
CV^a^	0.082^b^	0.016	0.020	0.017
Variance (SE)	0.048^b^	0.0019 (.0005)	0.0017 (.0005)	0.0016 (.0005)

^a ^It is equivalent to the relative inequality index √IM[2,2]. ^b ^log scale is applied in the calculation for the comparability with the model based measures.

The variation in parish level LE in this cohort was practically negligible. The analysis presented here is for illustration purposes rather than for substantive investigation.

In addition to the inequality measures in Table 3, parish means with their standard errors are estimated based on a gender combined model in which the reference category is made of 67 years old from middle household SES group residing in municipalities of average incomes. The estimated parish median LE of life remaining was 12.9 years (sd = 1.04, range: 12.0 ~ 13.4). The top and bottom ten parishes ranked by their median LE and standard error presented no substantial differences among parishes even for the extreme groups.

## Discussion

This study attempts to improve on both the individual-level approach and the aggregated analysis to assessing health inequality by using multilevel survival models to measure health inequalities in the life expectancy of an elderly Swedish cohort after 30 years of follow-up. The analysis on the large Swedish cohort data has shown that a multilevel log-duration model can be an improvement on existing methodologies in the study of health inequalities of life expectancy among individuals, within areas or across areas in a population, in several aspects. First, total variance in life expectancy can be decomposed explicitly into more than two components to measure multiple macro and micro level stochastic elements in health inequalities. Secondly, the complete specification of the distribution of life expectancy by individual covariates, such as gender or social group, is provided in the fixed effects part of the model like any regression model, which determines means of gender or social groups. The variance components of random effects for each subgroup can also be estimated in the same model. The model may be of interest to both social scientists whose research is focused on the mean differences of social position on individuals and to those who are interested in the variation of life expectancy within subgroups of a population. Thirdly, area discrepancy in life expectancy can be studied based on random effects at the area level with statistical uncertainty around each area mean. Differences in the characteristics of residences across areas can be adjusted for in the same model in order to achieve a better comparison between areas, which is more efficient than the traditional method of calculating life expectancy for each area separately to study area differences. Finally, the effects of interactions between covariates at different levels, such as municipality income and gender in our case, can be studied directly in the same model without resulting in biased estimates of standard errors. Similarly, joint effects of individual and area income on life expectancy of individuals can be studied readily in the analysis.

The proposed method can also be applied to data with a shorter period of follow-up. The main difference in data between the longer and shorter time of follow-up is that for the latter design there would be more individuals who were still alive and be considered as censored data by the time for data analysis, and the mortality rate could be too low. An alternative approach is to fit a multilevel proportional hazard model, or multilevel Cox model to fit hazard rate based on death or live indicator at each time point [23].

For many cross-sectional studies, health inequalities in mortality measure would be the main interest of researchers. Given impacts of individual and area socioeconomic factors on mortalities, multilevel logistic models have been widely used to study health inequalities [19,22,24]. The same analytic strategies presented in the present study can be applied.

The calculation of summary measures of absolute and relative health inequalities across individuals and across areas, an important area of interest to health policy makers, can be studied based on multilevel models. The study showed that, for subgroups of individuals, the model-based change pattern in coefficient variance of the specific relative health inequalities was consistent with that of the raw CV but also sensitive to the variables controlled in the model, and that the magnitude of the measure depends on the estimates of the mean and variance for each subgroup. Simple comparison of such model-based coefficients between countries of different datasets with different covariates in the analysis for each country may not be sensible. This problem can be overcome, however, by pooling data from all countries together in one model for the analysis. In the absolute measure of inter-individual differences, our model based measure II[1,1] showed a similar pattern to the Gini coefficient (based on ecological analysis for subgroups of individuals of different household SES), a similar pattern between gender, and less inequalities by the model based estimates than a conventional simple approach as expected. The main reason for the difference could be that the Gini coefficient was calculated using the unadjusted survival probability of the Kaplan-Meier (KM) estimator within each subgroup, while the model based II[1,1] was calculated from the survival probability of the fitted Model E after adjusting for other variables, as well as taking into account variation between households and between parishes in the model. The model based approach enables us to understand what macro-level or micro-level factors might be associated with, or accountable for, how much variation in the health measure of interest (life expectancy or mortality) and estimate comparable inequality measures at both individual and area levels after controlling for known factors or confounding.

The model-based Gini coefficient and the coefficient of variance across parishes as summary measures of inequalities in the LE among parishes appeared comparable to results from an ecological analysis. The between-parish differences in the LE could be compared graphically and numerically based on random effects across parishes. For small samples at the area level, simulation based methods such as MCMC are available for estimating the distribution and confidence intervals of random effects [16,18] for each parish.

Being focused on illustrating the methodology, this study used limited data which did not enable us to examine association between income at different levels or socio-economic position and inequalities in life expectancy in substantive depth. However, several important substantive findings have emerged in the study. Firstly, for this cohort most of the variation in remaining life expectancy after 1970 was among households and individuals. This suggests that in Sweden, where the socio-economic and health services tended to be distributed rather evenly among geographic areas, the health attributes and behaviours of household and individuals could be much more important in determining health outcome. However, this finding should not be generalised to other countries where wider regional social inequalities exist and cannot ignored. Multilevel modelling would be the adequate approach to analyse such data. Secondly, there were marked health inequalities among individual groups at different household socio-economic levels, especially within the poorest group, although household socio-economic differences explained only a small amount (1.8%) of variability in life expectancy at this level. Other factors such as ethnicity, lifestyle and morbidity rate of households could be further examined for their contribution to household health inequalities. Thirdly, comparable to other studies in the US and UK [15], males tended to have a larger coefficient of variation of life expectancy than females in this age group.

## Conclusion

Multilevel survival models for longitudinal observation of survival time with censored data can handle complex data with geographic structure or many levels of hierarchy. They have the flexibility and efficiency of estimating mean differences between subgroups of individuals with different characteristics, adjusting for confounding, explicitly quantifying variation of the outcome at different levels, and measuring impacts of covariates on variability of the outcome at various levels simultaneously in one single model. The model can be extended to survival data collected in small areas by directly pooling data together in a 2-level model weighted by the variance of the outcome variable of the individual to produce variance estimates of small areas for the comparison of any summary measures of health inequalities between areas [15,24]. The model illustrated can also be extended to an international model incorporating country as a separate level if many countries have small area health outcomes. If the outcome of interest is the risk of death or hazard rate instead of survival time or the LE, multilevel hazard models or multilevel discrete time models for death are readily applicable [25].

Much variability in life expectancy of individuals occurred at household and individual levels in this elderly Swedish cohort. Future research on determinants of health inequalities in the LE of the particular population should be focused on the household factors such as ethnicity, family life, contextual risks, health history of the family and individual factors such as genetics, socio-economic status, lifestyle, use of health services and health history etc. Further analysis on family and individual factors could reveal some important causes over the marked inequalities between individuals by their household poverty level reported in this study. Similar analysis on other age cohorts of the Swedish population could be interesting in establishing the magnitude of inequalities between age cohorts to identify possible intervention strategies to reduce such inequalities.

## Competing interests

The authors declare that they have no competing interests.

## Authors' contributions

JM and MY put together the specific aims of the study. JM provided the research database. MY performed data analysis and drafted the paper. SE and JM participated in the interpretation of the findings and helped to redraft in various versions. All authors read and approved the final manuscript.

## Supplementary Material

Additional file 1 **Table S1**. Fixed effects and variances of random effects (SE in brackets) for 3-level models, Skåne in Sweden, 1969-2000. Click here for file

Additional file 2 **Table S2**. Estimates of variation in life-expectancy among municipalities, parishes, households and individuals with varied level structure, Skåne in Sweden, 1969-2000. Click here for file
