# Supplementary material for: Multilevel survival analysis of health inequalities in life expectancy
Source: Int J Equity Health. 2009 Aug 23;8:31. doi: 10.1186/1475-9276-8-31 (PMC2740845; doi:10.1186/1475-9276-8-31)
Supplement: Additional file 1 — Table S1. Fixed effects and variances of random effects (SE in brackets) for 3-level models, Skåne in Sweden, 1969-2000. [file 1475-9276-8-31-S1.doc]

Table S1 - Fixed effects and variances of random effects (SE in brackets) for 3-level models, Skåne in Sweden, 1969-2000

|  | | | **Model A** | **Model B** | **Model C** | **Model D** | **Model E** |
| --- | --- | --- | --- | --- | --- | --- | --- |
| **Fixed Effects** | | | | | | | |
| Intercept | |  | 2.714 (.0060)§ | 2.039 (.0412)§ | 2.829 (.0088)§ | 2.328 (.0409)§ | 2.316 (.0409)§ |
| Gender | | Female | Reference |  | Reference | Reference | Reference |
|  | | Male | -0.323 (.0066)§ |  | -0.325 (.0066)§ | -0.328 (.0066)§ | -0.302 (.0073)§ |
| Age in 1970 | | 65 years |  |  | Reference | Reference | Reference |
|  | | 66 years |  |  | -0.057 (.0103)§ | -0.054 (.0102)§ | -0.055 (.0102)§ |
|  | | 67 years |  |  | -0.108 (.0104)§ | -0.107 (.0104)§ | -0.108 (.0104)§ |
|  | | 68 years |  |  | -0.183 (.0105)§ | -0.180 (.0104)§ | -0.181 (.0104)§ |
|  | | 69 years |  |  | -0.242 (.0106)§ | -0.238 (.0105)§ | -0.239 (.0106)§ |
| Household-SES | | Poorest |  | Reference |  | Reference | Reference |
|  | | Poor |  | 0.384 (.0443)§ |  | 0.342 (.0432)§ | 0.343 (.0432)§ |
|  | | Middle |  | 0.510 (.0413)§ |  | 0.498 (.0402)§ | 0.497 (.0403)§ |
|  | | Top |  | 0.584 (.0416)§ |  | 0.575 (.0406)§ | 0.578 (.0406)§ |
| Log-income of municipality  (centred at mean) | | |  |  |  |  | 0.021 (.0525) |
| Gender x log-income | | |  |  |  |  | -0.525 (.0620)§ |
| **Variance by Level** | | | | | | | |
|  | Parish | | 0.0019 (.0005)§ | 0.0017 (.0005)* | 0.0020 (.0005)§ | 0.0027 (.0006)§ | 0.0016 (.0005)* |
|  | Household | | 0.1150 (.0062)§ | 0.1166 (.0063)§ | 0.1108 (.0061)§ | 0.1088 (.0061)§ | 0.1105 (.0061)§ |
|  | Individual | | 0.5115 (.0036)§ | 0.5297 (.0037)§ | 0.5078 (.0035)§ | 0.5049 (.0035)§ | 0.5043 (.0035)§ |

* ***P<0.01***; ***§P<0.001***
