# Supplementary material for: Multilevel survival analysis of health inequalities in life expectancy
Source: Int J Equity Health. 2009 Aug 23;8:31. doi: 10.1186/1475-9276-8-31 (PMC2740845; doi:10.1186/1475-9276-8-31)
Supplement: Additional file 2 — Table S2. Estimates of variation in life-expectancy among municipalities, parishes, households and individuals with varied level structure, Skåne in Sweden, 1969-2000. [file 1475-9276-8-31-S2.doc]

##### Table S2 - Estimates of variation in life-expectancy among municipalities, parishes, households and individuals

##### with varied level structure, Skåne in Sweden, 1969-2000

|  | **2-level hierarchy** | | **3-level hierarchy** | | **3-level hierarchy** | | **4-level hierarchy** | |
| --- | --- | --- | --- | --- | --- | --- | --- | --- |
|  | **Variance (s.e.)** | **%** | **Variance (s.e.)** | **%** | **Variance (s.e.)** | **%** | **Variance (s.e)** | **%** |
| Municipality | 0.0362 (.0073)** | 6.4 | 0.0012 (.00046)* | 0.2 | 0.00083 (.00035)* | 0.1 | 0.00056 (.00037) | 0.09 |
| Parish |  |  | 0.0337 (.0045)** | 6.0 |  |  | 0.00106 (.00043)* | 0.2 |
| Household |  |  |  |  | 0.1101 (.0061)** | 17.8 | 0.1096 (.0061)** | 17.7 |
| Individual | 0.5275 (.0035)** | 93.6 | 0.5251 (.0035)** | 93.8 | 0.5084 (.0035)** | 82.1 | 0.5080 (.0035)** | 82.0 |

## Note: In all models age in 1970 and gender are included. Wald test for significance of variance estimates: *P<0.05; ** P<0.01
